# Depressive symptoms from kindergarten to early school age: longitudinal associations with social skills deficits and peer victimization

**DOI:** 10.1186/1753-2000-3-28

**Published:** 2009-09-21

**Authors:** Sonja Perren, Françoise D Alsaker

**Affiliations:** 1 Jacobs Center for Productive Youth Development, University of Zürich, Culmannstrasse 1, 8001 Zürich, Switzerland; 2 Department of Psychology, University of Berne, Muesmattstrasse 45, 3000 Bern 9, Switzerland

## Abstract

**Background:**

Depressive symptoms in children are associated with social skills deficits and problems with peers. We propose a model which suggests different mechanisms for the impact of deficits in self-oriented social skills (assertiveness and social participation) and other-oriented social skills (pro-social, cooperative and non-aggressive behaviors) on children's depressive symptoms. We hypothesized that deficits in self-oriented social skills have a direct impact on children's depressive symptoms because these children have non-rewarding interactions with peers, whereas the impact of deficits in other-oriented social skills on depressive symptoms is mediated through negative reactions from peers such as peer victimization.

**Method:**

378 kindergarten children (163 girls) participated at two assessments (Age at T1: M = 5.8, T2: M = 7.4). Teachers completed questionnaires on children's social skills at T1. Teacher reports on peer victimization and depressive symptoms were assessed at both assessment points.

**Results:**

Our study partially confirmed the suggested conceptual model. Deficits in self-oriented social skills significantly predicted depressive symptoms, whereas deficits in other-oriented social skills were more strongly associated with peer victimization. Longitudinal associations between other-oriented social skills and depressive symptoms were mediated through peer victimization.

**Conclusion:**

The study emphasizes the role of deficits in self-oriented social skills and peer victimization for the development of internalizing disorders.

## Background

Decades of research have shown that depressive symptoms in children are associated with social skills deficits and problems with peers [1-4]. Nevertheless, not much is known about the mechanisms underlying these associations. In the current paper a conceptual model is tested which tries to explain the interdependent effects of social skills and peer relations on the development of depressive symptoms.

Generally speaking social skills have been defined as behaviors that affect interpersonal relations [5]. In our work, we conceive of social competence as the ability to use social interactions to satisfy one's own goals and needs while at the same time considering the needs and goals of others. We differentiate between two dimensions: (a) *self-oriented social skills *which are aimed at satisfying one's own needs (e.g. assertiveness and social participation) and *other-oriented social skills *which are aimed at satisfying another's goals and needs (e.g. pro-social, cooperative and non-aggressive behavior) [6]. We assume that these dimensions operate through different mechanisms on depressive symptoms. First, we suggest that deficits in self-oriented social skills are directly associated with children's depressive symptoms because children's social needs and goals remain unsatisfied as a consequence of their inability to initiate social contacts, to express their needs, to assert themselves or to set limits to others' demands. These children therefore experience non-rewarding social interactions. Second, we propose that the impact of deficits in other-oriented social skills on depressive symptoms is mediated through negative peer relations, such as peer victimization.

### Social skills and depressive symptoms

Interpersonal theories of depression have tried to explain the observed associations between depression and relationship problems and deficits in social skills in children, adolescents and adults [7]. For example Lewinsohn [8] has suggested that a lack of certain social skills in depressed persons reduces their experience of positive reinforcement by others because they do not engage in behavior that leads to rewarding consequences. Coyne's theory [9] proposes that over the long term, a vicious circle develops: Depressive symptoms trigger negative reactions from others (e.g. aggressive behavior), which hinder recovery from depression. Moreover, intervention studies have shown that social skills training is an effective means to reduce depressive symptoms [4]. These latter results may even suggest that social skills play a causal role in the development of depression.

There is broad agreement that deficits in self-oriented social skills are associated with depressive symptoms. Withdrawal is considered as one of the main behavioral precursors of depressive symptoms in children [10] and it may exacerbate internalizing problems [11]. The empirical data on the association between deficits in other-oriented social skills and depressive symptoms are somewhat contradictory. Interestingly, aggressiveness - considered a deficit in other-oriented social skills - is positively associated with self-oriented social skills, i.e. aggressive children are more assertive and more prone to engage in social interactions [12-14]. Therefore, aggressiveness might even protect children from depressive symptoms. However, the specific role of pro-social behavior in the development of psychopathology has received only limited attention. It has been suggested that low levels of pro-social behavior may place children at risk for externalizing problems, whereas high levels of pro-social behavior might be a risk factor for internalizing problems [15-17]. It might be that a lack of pro-social behavior could be considered an indicator of a deficit in other-oriented skills, whereas high levels of pro-social behavior could also reflect a lack of self-oriented skills because these children might be too considerate of the needs of others and neglect their own feelings and needs [18]. In fact, cross-sectional and longitudinal studies have found that children who are overly concerned for the welfare of others, are highly cooperative or over-friendly, have elevated levels of emotional symptoms [19-21]. Perren and collaborators showed that at kindergarten age pro-social behavior predicted increases in emotional symptoms, but only in children who already had emotional problems at the first assessment point [18]. On the other hand, some studies found pro-social behavior to be negatively associated with emotional symptoms [22,23]. Pro-social behavior was also shown to be a protective factor in terms of peer acceptance in children with emotional symptoms [24]. Therefore, considering simultaneously the impact of self- and other-oriented social skills on depressive symptoms might give further insights into these apparently controversial results.

### Social skills and peer victimization

Deficits in certain social skills may lead to negative reactions by peers, such as rejection or victimization. Peer rejection may be a precursor of peer victimization [25] and may play a crucial role in stabilizing a child's victim role [26]. In fact Ladd and Troop-Gordon [27] reported peer rejection to be predictive of later victimization and victimization of later rejection.

Peer rejection has consistently been associated with aggressive and withdrawn behavior [28], i.e. with deficits in other- and self-oriented social skills. Likewise, in bully/victim research two different pathways to victimization are suggested and the need to differentiate between two types of victims has been emphasized: (1) children who are aggressive and victimized (aggressive victims) and (2) children who are victimized without being aggressive (passive victims) [12,14,29,30]. Accordingly, aggressive as well as withdrawn-submissive behavior patterns are related to peer victimization.

Submissiveness has been discussed as a hallmark of victimization. One explanation for this association is that bullies are looking for easy targets for their assaults [31]. Several studies revealed that passive victims have problems defending themselves [14,32] and that they are less assertive, for example using fewer persuasion attempts [33]. However, Perren et al. [6] could not confirm submissiveness as being an overall predictor of peer victimization in kindergarten children and the study by Fox and Boulton [34] reported submissive behavior in school children to be longitudinally predictive of social exclusion only. Withdrawal behavior (also reflecting a deficit in self-oriented skills) may be associated with victimization by (1) suggesting vulnerability, (2) suggesting low risk of retaliation, and (3) hindering children to find supporting and protecting friends in the class [35]. Also, withdrawn children are not salient and socially less rewarding for their peers. This, in itself, might lessen the chance that peers would help them when they become victimized.

However, in younger children, aggressive behavior seems to be a stronger predictor of victimization and rejection than withdrawn-submissive behavior [36-38]. Nevertheless, not all aggressive children are at risk for becoming victimized and different findings suggest that the most important difference between non-victimized aggressive children (bullies) and aggressive victims consists in their respective ability or inability to control their physical aggression. These uncontrolled aggressive children are fairly disturbing in the class and it seems rather obvious that peers could easily be influenced to assist the bullies [35].

In sum, deficits in self- and other-oriented social skills are associated with rejection and victimization and deficits in other-oriented social skills have been found to be stronger predictors than deficits in self-oriented social skills.

### Peer victimization and depressive symptoms

Children with depressive symptoms have generally been reported to have poorer peer relations in terms of popularity, rejection or victimization [22,39,40]. Hawker and Boulton's [41] meta-analysis of cross-sectional associations between peer victimization and psychosocial maladjustment showed that victimization is most strongly related to depression and least strongly to anxiety. Peer victimization and exclusion may also increase children's depressive symptoms [11,42-44] or even be causally related to the development of self-derogation and depressive problems [45,46]. Peer victimization is also associated with health problems, suicidality, and poor school adjustment [47-50].

In sum, empirical findings consistently show that depressive symptoms are associated with negative peer relations and that peer rejection and victimization may play a causal role in the development of depressive symptoms.

### The interplay of social skills, peer victimization, and depressive symptoms

As shown above, social skills deficits are not only associated with depressive symptoms but are a strong predictor of peer victimization. Therefore, we assume that these variables interact in systematic ways, especially when we differentiate between self-oriented and other-oriented skills.

As outlined by Bukowski and Adams [51], peer relations have been discussed as markers, mediators, or moderators for maladjustment in children and adolescents. Similarly, Ladd [52] suggested different "Child by Environment Models" which take into account the interplay between child behavior, peer relations, and the development of internalizing (and externalizing) disorders. Empirical results mainly support additive or mediation models. For example a four-year longitudinal study by Ladd [10] has provided support for the additive model. Withdrawn behavior and peer rejection were shown to be overlapping risk factors for the development of children's internalizing problems. A study by Dill and collaborators demonstrated that peer rejection and victimization mediate between children's withdrawn/shy behavior and negative affect [53]. Similarly, a study among young adults showed interpersonal relationships to mediate the impact of social skills on well-being [54]. The conceptual model to be tested in the current paper was partly tested in a cross-sectional study with 198 kindergarten children [6]. The study confirmed the distinct contribution of self- and other-oriented social skills on children's peer victimization and emotional well-being. Deficits in self-oriented social skills predicted higher levels of emotional symptoms, whereas deficits in other-oriented social skills predicted higher levels of peer victimization. The suggested mediating role of peer victimization was partly confirmed.

In the present paper we aim to replicate the findings in a larger sample and most importantly, to include longitudinal data. We hypothesize that deficits in self- and other-oriented social skills are associated with both peer victimization and depressive symptoms. Furthermore, we hypothesize that the impact of deficits in other-oriented social skills on depressive symptoms is mediated through peer victimization whereas deficits in self-oriented social skills are directly associated with depressive symptoms. We also hypothesize that peer victimization is associated with an increase in depressive symptoms over time. We will examine whether the associations are moderated by the child's gender.

In our study we are adopting a dimensional approach of assessing depressive symptoms [55], i.e. we do not use clinical diagnoses of depression.

## Method

### Study design and sample

The data come from a large longitudinal study of pathways to peer victimization in a representative sample of kindergarten and elementary school children in the German-speaking part of Switzerland [56]. Written parental consent was obtained for all participants. Children themselves gave oral assent prior to the first interview and knew they could withdraw from the study at any time.

Due to organizational reasons the original recruiting was lagged (T1 assessment at two consecutive school years) but the follow-up was conducted in the same school year. Therefore, the time interval between the two assessments ranges between one and two years; and children of subsample 1 are older than those of subsample 2 at Time 2. Thus, we control for subsample membership in all analyses. Some kindergarten groups also participated in a prevention program against bullying. Thus, we reran all analyses reported in this paper, including intervention participation as a control variable. None of the reported results showed any change. Thus, in the final analyses this variable was not included. Only children with valid data at both assessment points are included in the current paper.

378 children (163 girls) participated (Age_T1: M = 5.8, SD = 0.6, Age_T2: M = 7.4, SD = 0.8). At T1 35% children were in the first year and 65% in their second year of kindergarten (age-mixed groups). At T2, most children were in the first grade of primary school (54%), 24% in second grade and 22% were in their second year of kindergarten.

Children were primarily white and German speaking, but there is large proportion of children with an immigrant background: 26% of participating children have both parents and 16% one parent with a non-Swiss country of origin. These percentages correspond with general population statistics.

### Instruments

Teachers completed questionnaires on a range of dimensions covering children's psychosocial adjustment and social behavior. Teachers rated children's peer victimization and depressive symptoms at Time 1 and Time 2, social skills were only assessed at Time 1. Children's behaviors were rated by different teachers at Time 1 and at Time 2. Prior to data collection at T1, teachers were offered a workshop during which they received in-depth information about bullying and victimization (each class was represented by at least one teacher). Teachers who completed the questionnaires at T2 did not participate in this workshop.

#### Peer victimization

Teachers rated each child on four victimization items (physical, verbal, object-related, exclusion; e.g. 'child is victimized verbally, i.e., laughed at, called names, teased.') on a 5-point rating-scale (never, seldom, once or several times a month, once a week, or several times a week [14,57]). The four items yielded an adequate reliability coefficient (T1: α = .81; T2: α = .85). For the purpose of the present research question, a mean score of the four items was used.

#### Depressive symptoms

The scale consists of three items. Two items were selected from the German version of the Child Behavior Checklist/TRF [58]("He/she seems to be unhappy, sad"; "He/she speaks pejoratively about him/herself") and one item was added that was very similar to the first item ("He/she looks a little sad") in order to increase the scale's reliability. This item was taken from the list of typical symptoms for children aged 3-6 years according to the German guidelines for the diagnosis of psychological disturbances in infants, children and adolescents [59,60] All three items were rated on a four-point scale (0 = not at all true to 4 = definitely true) and yielded adequate reliability (T1: α = .71, T2: α = .76).

#### Social skills

Teachers completed a questionnaire on children's social behavior (SOCOMP; [61]) The two dimensions self- versus other-oriented social skills were built using a combination of six subscales of social behavior patterns All items were rated on a four-point scale (0 = not at all true to 4 = definitely true). First, six different social skills scales were computed. The *cooperative behavior *subscale consists of five items (e.g. "Compromises in conflicts with peers"; α = .79). The *pro-social behavior *subscale consists of five items, covering helping, comforting, and sharing behavior (e.g. "Frequently helps other children"; α = .86). The *overt aggression *subscale (physical and verbal) consists of 4 items (e.g. "Kicks, bites or hits other children"; α = .88). The *social participation *subscale, covering propensity to participate in social interactions, consists of 4 items (e.g. "Converses with peers easily", α = .82). The *leadership *subscale consists of three items ("organizes, suggests play activities to peers"; α = .82). The *setting limits *subscale also consists of three items (e.g. "Refuses unreasonable requests from others"; α = .76).

#### Validation of the two-dimensionality of social skills

A principal component analysis was conducted to validate the suggested dimensions of self- and other-reported social skills. The six social behavior subscales were entered in the analysis (PCA with Varimax-Rotation, Eigenvalue > 1 is used as criterion). In accordance with the hypothesized dimensions the analysis yielded two factors. The first factor (explained variance 41%) had high factor loadings for the subscales cooperative behavior (.90), pro-social behavior (.81) and overt aggression (-.85). The second factor (explained variance 34%) had high factor loading for the subscales sociability (.85), leadership (.91) and setting limits (.81). All cross-loadings were <.33. Based on this analysis, two dimensions of social skills were computed. The scale *other-oriented social skills *is the mean of the z-standardized subscales cooperative behavior, pro-social behavior and overt aggression (reversed). The scale *self-oriented social skills *is the mean of the z-standardized subscales social participation, leadership and setting limits.

### Overview of the statistical analyses

For the bivariate associations Pearson correlations were used, for the multivariate analyses several multiple regression analyses were computed (all predictors were entered simultaneously and remained in the final model).

#### Mediation

To test for mediation, we adopted the commonly used approach by Baron and Kenny [62]. This mediation analysis consists of three steps. In a first step, we investigated the impact of social skills on peer victimization. In step 2, we investigated the impact of social skills on depressive symptoms. In step 3, we analyzed whether peer victimization mediates the associations between social skills and depressive symptoms. A variable is then considered to mediate when the effect of the first predictor drops to zero, when controlling for the mediator variable.

#### Changes over time

For each of the above mentioned steps, two regression models were computed. First the effect of the control variables and social skills at T1 was tested for T2 outcomes. In a second step the score of the outcome measures at T1 was entered as additional independent variable in order to predict changes in the outcome variable over time [63].

#### Gender as moderator

As we were interested whether gender moderates the associations we also included the interaction effects. Gender was dummy coded (boys = 0, girls = 1). All analyses were first computed without the gender interaction and than including the gender interaction. As the results remained largely the same, only the results including the interactions are presented below.

## Results

Bivariate correlations between all study variables are shown in Table 1. The two dimensions of social skills were not significantly associated. Deficits in other-oriented skills were significantly associated with depressive symptoms and victimization at both assessment points. Deficits in self-oriented skills were significantly associated with depressive symptoms (T1 and T2), and victimization (T1 only). Depressive symptoms and victimization were significantly associated at both assessment points. Furthermore, depressive symptoms and peer victimization were moderately stable from T1 to T2.

**Table 1:** Bivariate associations between main study variables (Pearson correlations)

	**SOS**	**Dep T1**	**Dep T2**	**Vict T1**	**Vict T2**	**Age**	**Gender (girls = 1)**
Other-oriented social skills (T1)	.09	-.25**	-.18**	-.46**	-.30**	.15**	.23**
Self-oriented social skills (T1)		-.48**	-.23**	-.18**	.01	.24**	.14**
Depressive symptoms (T1)			.35**	.29**	.12*	.01	-.05
Depressive symptoms (T2)				.15**	.30**	.01	-.13*
Victimization (T1)					.26**	.02	-.21**
Victimization (T2)						-.03	-.22**

** p < .01, *p < .05 (two-tailed)

Age was positively associated with both dimensions of social skills. In addition, girls showed higher levels of other- and self-oriented social skills and lower levels of victimization (T1 and T2) and depressive symptoms (T2) than boys.

### Social skills predicting peer victimization (Step 1)

In a first set of regression analyses we investigated the impact of social skills on victimization at T2. Gender, age and subsample membership (see method section) were entered as control variables. In a second step victimization at T1 was entered as additional independent variable in order to predict changes in victimization over time. The analyses yielded significant effects of gender and other-oriented social skills on level of victimization at T2 and changes in victimization (see Table 2). Boys were more frequently victimized than girls, and also showed a larger increase in victimization over time. The lower the level of other-oriented social skills, the higher the level of peer victimization, and also the greater the increases in peer victimization over time.

**Table 2:** Results for the regression analyses predicting peer victimization and depressive symptoms at T2 (betas)

	**STEP 1**		**STEP 2**		**STEP 3**	
	**Victimization (T2)**	**Victimization (Change^b^)**	**Depressive symptoms (T2)**	**Depressive symptoms (Change^b^)**	**Depressive symptoms (T2)**	**Depressive symptoms (Change^b^)**
Gender^a^	-.162**	-.153**	-.059	-.078	-.024	-.052
Age (T1)	.006	.019	.090	.044	.082	.042
Sample	-.070	-.053	-.012	.001	-.001	.005
Other-oriented social skills (T1)	-.304***	-.240***	-.209**	-.148*	-.117	-.083
Self-oriented social skills (T1)	.053	.075	-.248**	-.104	-.251***	-.121
Gender*OOS^a^	.064	.049	.079	.089	.044	.060
Gender*SOS^a^	-.013	-.017	.023	.023	.007	.008
Victimization (T1)	--	.125**	--	--	.047	.011
Victimization (T2)			--	--	.217***	.202**
Gender*VIC (T1)^a^			--	---	-.202	-.196
Gender*VIC (T2)^a^			--	--	.202	.200
Depressive symptoms (T1)				.285***		.268***
Model: R^2^	.126	.137	.094	.131	.167	.190

*** p < .001 ** p < .01, *p < .05

^a ^Dummy coded variables (grls = 1, boys = 0)

^b ^The outcome at T2 were used as dependent variable but controlled for its level at T1, thus we predict changes from T1 to T2 (positive scores = increases).

### Social skills predicting depressive symptoms (Step 2)

Following the same procedure as presented above, we examined the impact of social skills on depressive symptoms. Results are shown in Table 2.

The control variables gender, age and sample had no significant effect on level and change of depressive symptoms. No significant interactions with gender emerged. The analyses yielded significant effects of both dimensions of social skills on depressive symptoms. Children with lower levels of other-oriented social skills and self-oriented social skills at T1 had higher levels of depressive symptoms at T2. Deficits in other-oriented social skills also predicted increases in depressive symptoms.

### Peer victimization as a potential mediator (step 3)

To analyze the potential mediating effects of peer victimization, the variable was entered at the third step of the regression analyses [62]. In this analysis, peer victimization at T1 and T2 were entered as independent variables (including gender interactions). Victimization at T2 was a significant predictor of depressive symptoms at T2 and also predicted increases in depressive symptoms over time (see Table 2). When controlling for peer victimization, the effect of self-oriented social skills remained unchanged. That is, self-oriented social skills predicted depressive symptoms at T2, but no increases in depressive symptoms. In contrast, when controlling for peer victimization at T2, the significant associations between other-oriented social skills and depressive symptoms at T2 disappeared. The same effect was shown in regards changes in depressive symptoms.

## Discussion

Our study has shown that self- and other-oriented social skills are independent dimensions, and that both are directly or indirectly associated with depressive symptoms.

### The role of self-oriented social skills

We hypothesized that deficits in self-oriented skills could make a child an easy target for bullies. The hypothesis received some support, but the association was relatively weak, in the multivariate analyses the significant effect even disappeared. This finding confirms that in preschool and kindergarten age submissive-withdrawn behaviors are less strong predictors for peer victimization than aggressive behaviors [6,38]. Victimization in itself was also a strong predictor of depressive symptoms but it did not act as a mediator regarding self-oriented social skills. That is, both deficits in self-oriented skills and victimization predict depressive symptoms in young children. This is in line with previous studies. The association between victimization and depressive symptoms has been repeatedly documented, both in school-age and kindergarten children [64,65], and the effect of victimization on depressive symptoms has been shown to persist into early adulthood, long after victimization had stopped [66].

It still remains to elucidate which processes might link self-oriented social skills with depressive tendencies. It might be that children who have difficulties in setting limits or asserting themselves seldom experience the satisfaction of their own wishes and needs. They might therefore experience relatively little reward in social situations and see themselves as incompetent in social contexts. Knowing that their chances to achieve their social goals are modest, they might gradually take less initiative in the peer group, and thus also become less attractive to their peers. The quality of their peer relations might therefore become highly dependent on the personality and behavior of their peers. In other words, they might perceive themselves as low in self-efficacy and highly dependent on their peers' moods. Knowing how important positive peer relations are to children's well-being [67], it seems plausible that deficits that prevent children from developing rewarding relations with their peers might lead at least to sadness and possibly to other depressive symptoms. There might also be a reciprocal relationship between lack of social skills and depressive symptoms [2]. On the one hand, being sad and depressed or being victimized may result in further withdrawal from peers, i.e. unsociability may turn to peer avoidance [68]. A lack of experience with peers may in turn decrease children's social skills as they miss important learning opportunities. In fact, it has been shown that depressive symptoms lead to a decrease in self-evaluated competence [69].

### The role of other-oriented social skills

Considering deficits in the second dimension of social skills examined in our study, other-oriented skills, we found a mediating effect of victimization. Other-oriented skills were operationalized in terms of pro-social and cooperative behavior and non-aggressiveness. In other words, children low in other-oriented skills were typically high in aggressive behavior and low in behaviors such as sharing, helping and being cooperative. These are all characteristics of aggressive victims [35]. In fact, our results show that deficits in other-oriented social skills were associated with victimization. Most importantly, the association between depressive symptoms and this type of deficit disappeared when victimization was entered in the regression analysis. This is perfectly in line with earlier studies on bully/victim problems that have demonstrated that bullies (who are defined as being aggressive and not victimized by their peers) do not suffer from internalizing problems. That is, deficits in other-oriented social skills, that are typical both for bullies and aggressive victims, do not automatically lead to depressive symptoms. They only do so when the children are experiencing victimization by their peers.

### Gender differences

The study yielded several gender differences, but gender did not moderate the associations between social skills, victimization and depressive symptoms. Boys were more frequently victimized than girls. This goes in line with studies showing that boys are more frequently aggressive victims (but not passive victims) than girls [14]. In correspondence with other studies [15], girls were more prosocial-cooperative and less aggressive than boys.

In our study we did not differentiate between different forms of victimization. However, some studies have shown that boys and girls may not be victims of the same types of aggression, e.g. preschool boys were more frequently victimized physically than girls [70]. Further studies have to show whether different types of social skills deficits are associated with different forms of victimization.

### Strengths and limitations of the study

Results are based on data from a rather large representative sample of kindergarten and primary school children. In the current paper, only data from children who participated also in the follow-up were included. Attrition was mainly due to the unwillingness of school principals or teachers who had not participated in the first wave of data collection, i.e. there was no systematic attrition with respect to children's or families' characteristics. Therefore, we do not expect systematic biases due to attrition. However, our results may be somewhat biased because we did not control for other potentially important variables such as family background or children's cognitive or verbal abilities [71,72].

One important limitation of our study is that we only used a three-item measure to assess children's depressive symptoms. Due to the small number of items, other potential symptoms for depression (e.g. lack of energy, hopelessness, psychosomatic complaints, preoccupation with death) were not assessed. Further studies have to show whether lack of assertiveness and sociability and peer victimization are merely associated with low emotional well-being or depressed mood or in fact are predictive for more severe cases of childhood depression.

In our conceptual model we assume that deficits in social skills precede children's depressive symptoms and peer victimization. However, social skills were only assessed at the first measurement point. Therefore, we could not analyze whether depressive symptoms or peer victimization predict changes in social skills.

In the current study we only included teacher reports; therefore we have shared method variance in our data. Regarding the validity of teacher reports on depressive symptoms, we relied on the strong tradition of the Achenbach questionnaires which reliably can be completed by parents or teachers. Studies have shown a moderate agreement between teacher, parent and self reports regarding children's depressive symptoms [40,73,74] indicating that teachers can provide valid reports on children's depressive symptoms. Nevertheless, the inclusion of other informants might give even more reliable and valid data [75]. The inclusion of children's self-perceptions, e.g. of depressive symptoms, may be particularly important as children have their own meaningful perspective on their experience [40]. As to victimization, an earlier study revealed a high degree of correspondence between teacher reports and peer nominations in kindergarten [14].

### Clinical implications

Our model suggests different intervention strategies considering both improvements in children's social skills and their peer relations. Until now, most (universal) prevention and intervention strategies in children focus on the promotion of empathy, pro-social behavior, and reduction in aggressive behavior [76]. Our results suggest that prevention and intervention efforts in children should also consider the promotion of self-oriented social skills, i.e. efforts to improve children's social participation and assertiveness. Training specifically aimed at improving children's self-oriented social skills, i.e. their social initiative and assertiveness, might even be considered as a psychotherapeutic approach to childhood depression. Shifting the focus toward self-oriented social skills is also important, as studies have shown that for some children being highly pro-social or empathic may even be negative for their own emotional well-being [18]. Prevention approaches should aim at establishing a balance between both dimensions of social skills, i.e. to promote children in their ability to use social interactions to satisfy their own goals and needs while at the same time considering the needs and goals of others.

## Conclusion

Our study confirmed the suggested conceptual model. Deficits in self-oriented social skills significantly predicted depressive symptoms, whereas deficits in other-oriented social skills were more strongly associated with peer victimization. Longitudinal associations between other-oriented social skills and depressive symptoms were mediated through peer victimization. We suggested that the direct link between deficits in self-oriented social skills and depressive symptoms might partly be mediated by non-rewarding interactions with peers. More specific research is needed on these potential mediating mechanisms, for example in terms of socio-cognitive or socio-emotional processes. The study emphasizes the role of deficits in self-oriented social skills and peer victimization for the development of internalizing disorders.

## Competing interests

The authors declare that they have no competing interests.

## Authors' contributions

SP was responsible for the conceptual background of the paper, analyzed and interpreted the data and drafted the manuscript. FA is grant-holder, conceived and directed the study, and was actively involved in writing up the manuscript. Both authors read and approved the final manuscript.
